# Effect of Intensive Blood Pressure Lowering on the Risk of Incident Silent Myocardial Infarction: A Post Hoc Analysis of a Randomized Controlled Trial

**DOI:** 10.1111/anec.70018

**Published:** 2024-10-03

**Authors:** Richard Kazibwe, Muhammad Imtiaz Ahmad, Sanjay Singh, Lin Y. Chen, Elsayed Z. Soliman

**Affiliations:** ^1^ Department of Internal Medicine Wake Forest School of Medicine Winston‐Salem North Carolina USA; ^2^ Department of Internal Medicine, Section on Hospital Medicine Medical College of Wisconsin Milwaukee Wisconsin USA; ^3^ Lillehei Heart Institute and Cardiovascular Division University of Minnesota Medical School Minneapolis Minnesota USA; ^4^ Epidemiological Cardiology Research Center (EPICARE), Department of Internal Medicine, Cardiovascular Section Wake Forest School of Medicine Winston‐Salem North Carolina USA

**Keywords:** blood pressure control, silent myocardial infarction, SPRINT

## Abstract

**Background:**

Silent myocardial infarction (SMI) frequently goes undetected, yet it is associated with increased cardiovascular morbidity and mortality. The impact of intensive systolic blood pressure (SBP) lowering on the risk of SMI in those with hypertension remains uncertain.

**Methods:**

In this post hoc analysis of the Systolic Blood Pressure Intervention Trial (SPRINT), participants with serial electrocardiograms (ECGs) during the trial were included. SPRINT investigated the benefit of intensive SBP lowering, aiming for < 120 mmHg compared to the standard SBP goal of < 140 mmHg. Incident SMI was defined as evidence of new MI on an ECG without adjudicated recognized myocardial infarction (RMI).

**Results:**

During a median follow‐up of 3.9 years, a total of 234 MI events (55 SMI and 179 RMI) occurred. Intensive, compared to standard, SBP lowering resulted in a lower rate of SMI (incidence rate 1.1 vs. 2.3 cases per 1000 person‐years, respectively; HR [95% CI]: 0.48 [0.27–0.84]). Similarly, intensive, compared to standard, BP lowering reduced the risk of RMI (incidence rate 4.6 vs. 6.5 cases per 1000 person‐years, respectively; HR [95% CI]: 0.71 [0.52–0.95]). No significant differences were noted between the strength of the association of intensive BP control on lowering the risk of SMI and RMI (*p*‐value for HR differences = 0.23).

**Conclusions:**

This study shows that in adults with hypertension, the benefits of intensive SBP lowering, compared with standard BP lowering, go beyond the prevention of RMI to include the prevention of SMI.

**Trial Registration:**

ClinicalTrials.gov Identifier: NCT01206062.

## Graphical abstract

In this post hoc analysis of the SPRINT trial, intensive systolic blood pressure (SBP) lowering in individuals with high‐risk hypertension was associated with a reduced the risk of ECG‐based silent myocardial infarction (MI) compared to standard SBP lowering. This demonstrates additional benefits of intensive SBP treatment beyond preventing clinically recognized MI.

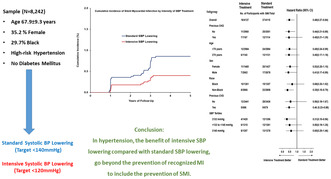

## 1 Introduction

According to a report by the American Heart Association (AHA), annually, 805,000 first and recurrent myocardial infarctions occur, with approximately 170,000 events being unrecognized or silent myocardial infarctions (SMI) (Tsao et al. 2023), and individuals with SMI had worse outcomes compared to recognized MI (RMI) in some studies (Soliman 2019). Individuals with RMI benefit from effective secondary preventive therapies to reduce this the risk of further cardiovascular events and mortality (Nogami et al. 2022). However, failure to promptly detect SMI represents a missed opportunity to initiate these preventive therapies. Consequently, individuals with SMI remain susceptible to adverse events (Nogami et al. 2022).

Hypertension is a major modifiable risk factor for ischemic heart disease, and clinical trials have shown approximately a 15%–25% reduction in the risk of MI with effective blood pressure (BP) control (Chobanian et al. 2003). In the final report of the Systolic Blood Pressure Intervention Trial (SPRINT), intensive SBP control of less than 120 mmHg, compared to the standard goal of SBP of 140 mmHg, resulted in a 28% relative risk reduction of MI (SPRINT Research Group et al. 2021). The adjudication of MI as a secondary endpoint in SPRINT included a combination of clinical RMI and SMI ascertained from electrocardiograms (ECG) (Ambrosius et al. 2014). Randomized controlled trials (RCTs) often include SMI in the definition of MI as a clinical endpoint to enhance statistical power, reduce sample size, and consequently, shorten the duration of the trial (Dahlöf et al. 2005; Marso et al. 2016). However, it is unknown if the effect of intensive BP control strategy would reduce the development of SMI. Therefore, we conducted a secondary analysis of the SPRINT with the hypothesis that intensive BP lowering would reduce the incidence and risk of SMI when compared to the standard BP control.

## 2 Methods

### 2.1 Design and Sample

The rationale and design of the SPRINT trial have been previously reported in detail (Ambrosius et al. 2014) and a copy of the protocol is available in Figure S2. The CONSORT diagram for the study is shown in Figure S2. Briefly, SPRINT was a randomized, controlled, open‐label trial in which 9361 participants ≥ 50 years of age with SBP ≥ 130–180 mmHg and at high risk for or with CVD were randomized to achieve either an SBP target of < 140 mmHg (standard treatment group) or < 120 mmHg (intensive treatment group). SPRINT aimed to test whether intensive SBP control to < 120 mmHg reduces a composite of CVD events. High CVD risk was defined as ≥ 1 of the following: clinical or subclinical CVD, chronic kidney disease (CKD), 10‐year risk of CVD ≥ 15% by Framingham risk score, or age ≥ 75 years. A detailed protocol of the SPRINT trial has been previously published (Ambrosius et al. 2014). The Institutional Review Board approved the SPRINT study at each participating study site, and all participants provided written informed consent.

In this analysis, we included SPRINT participants with at least one follow‐up ECG, thereby excluding 551 and 568 participants from the intensive and standard arm, respectively, due to missing follow‐up ECGs. A de‐identified dataset was obtained from the National Heart, Lung, and Blood Institute's Biologic Specimen and Data Repository Information Coordinating Center after the institutional review board at the Medical College of Wisconsin approved the study.

### 2.2 Study Outcomes

Clinical and laboratory data were obtained at baseline and every 3 months for the first year, then every 6 months. Data regarding potential outcomes were assessed every 3 months using a structured interview to minimize ascertainment bias, and a standard protocol, centralized monitoring by the coordinating center, was used to obtain relevant information, including medical records, laboratory reports and ECGs (Ambrosius et al. 2014). RMI was ascertained from the adjudication of hospital records for clinical events using cardiac symptoms, biomarkers and ECG criteria. SMI, using 12‐lead ECG at years 2 and 4 and the close‐out visit compared to baseline, was determined centrally as a finding of a new significant Q wave in the absence of clinical RMI using the Minnesota ECG classification (SPRINT Research Group et al. 2021). A Morbidity and Mortality committee formally adjudicated all clinical events, including MI, using a standardized electronic form. Adjudicators were blinded to treatment assignment (Ambrosius et al. 2014).

### 2.3 Statistical Analysis

Baseline characteristics were compared among participants who did or did not experience SMI during the trial. Continuous variables were presented as mean, standard deviation, and categorical variables as numbers with percentages.

Time until the first occurrence of SMI and RMI was compared between the two treatment arms using the intention‐to‐treat approach for all randomized participants. We used Cox proportional hazards regression with 2‐sided tests at the 5% significance level, with stratification by randomization site. Follow‐up time was censored on the date of the last event ascertainment. Interactions between treatment arm and prespecified subgroups (age [< 75 vs. ≥ 75 years], sex, race [black vs. nonblack], SBP tertiles [≤ 132, > 132 to < 145, ≥ 145 mmHg], prior cardiovascular disease, and prior chronic kidney disease [defined as an estimated glomerular filtration rate of < 60 mL per min per 1.73 m^2^ of body‐surface area]) were assessed with a likelihood ratio test for interaction. Because the proportional hazards assumption was violated with the RMI as an outcome, we split the maximum follow‐up time of approximately 5 years and compared time to occurrence of MI between treatment arms in the short‐term (within 2.5 years follow‐up) and long‐term (beyond 2.5 years follow‐up). We compared the association of intensity of SBP treatment with SMI and RMI, applying the competing risk approach proposed by Lunn and McNeil (Lunn and McNeil 1995), applied to an augmented data set. All statistical analyses were performed using SAS version 9.4 (SAS Institute Inc., Cary, NC).

## 3 Results

In this analysis included 8242 participants (age, 67.9 ± 9.3 years, 35.2% women, and 29.7% black) from the SPRINT trial. Of these 4127 were assigned to the intensive SBP treatment arm and 4115 were assigned to the standard SBP treatment arm. SPRINT participants were followed for up to 5.4 years (median, 3.9 years). Table 1 shows the baseline clinical characteristics for the cohort based on no MI, and RMI or SMI occurrence during the trial. Compared to participants with RMI, those with SMI were more likely to be female, report no prior history of CVD, and have lower Framingham CVD risk scores, but had higher diastolic BP and HDL‐C levels than those with RMI.

**TABLE 1:** Baseline characteristics by myocardial infarction among 8242 SPRINT participants.

Characteristic mean ± SD, *n* (%) or median (IQR)	No MI (*N* = 8008)	RMI (*N* = 179)	SMI (*N* = 55)	*p* ^a^	*p* ^b^
Age, years	67.0 ± 9.3	71.6 ± 8.9	70.0 ± 9.2	0.252	< 0.001
Age ≥ 75 years	2180 (27.2)	75 (41.9)	19 (34.5)	0.331	< 0.001
Female	2828 (35.3)	43 (24.0)	31 (56.3)	< 0.001	< 0.001
Prior CKD	2195 (27.4)	67 (37.4)	19 (34.6)	0.698	0.006
Prior CVD	1546 (19.3)	76 (42.5)	15 (27.3)	0.043	< 0.001
Framingham 10‐year CVD risk score	17.5 ± 10.6	26.4 ± 13.5	19.6 ± 11.4	0.001	< 0.001
Framingham risk ≥ 15%	4893 (61.1)	135 (75.4)	29 (52.7)	0.001	< 0.001
Race or ethnicity group					
Non‐hispanic black	2390 (29.9)	31 (17.3)	23 (41.8)	0.002	0.003
Hispanic	838 (10.5)	17 (9.5)	3 (5.5)		
Non‐hispanic white	4639 (57.9)	128 (71.5)	28 (50.9)		
Other	141 (1.8)	3 (1.7)	1 (1.8)		
Current smoker	1010 (12.6)	35 (19.6)	12 (21.8)	0.714	0.003
BMI, kg/m^2^	29.1 ± 5.7	28.9 ± 5.1	29.4 ± 6.5	0.562	0.059
Systolic BP, mmHg	138.0 ± 15.5	140.9 ± 15.4	141.6 ± 16.7	0.762	0.300
Diastolic BP, mmHg	78.0 ± 11.8	74.5 ± 13.5	79.7 ± 12.8	0.012	< 0.001
SBP tertile					
≤ 132 mmHg	2705 (33.8)	55 (30.7)	16 (29.1)	0.814	0.790
> 132 to < 145 mmHg	2609 (32.6)	64 (35.8)	18 (32.7)		
≥ 145 mmHg	2694 (33.6)	60 (33.5)	21 (38.2)		
eGFR, mL/min per 1.73 m^2^	71.5 ± 20.3	67.1 ± 20.8	68.1 ± 20.1	0.759	0.002
Creatinine, mg/dL	1.0 ± 0.3	1.1 ± 0.4	1.09 ± 0.36	0.377	0.007
UACR	9.4 (5.6–20.6)	14.9 (6.8–41.4)	12.1 (6.3–27.3)	0.278	< 0.001
Total cholesterol	187.0 ± 41.1	185.9 ± 43.4	188.5 ± 45.7	0.699	0.366
HDL, mg/dL	50.0 ± 14.4	49.2 ± 12.5	55.4 ± 14.5	0.002	0.001
Triglycerides, mg/dL, median	106 (77–150)	122.0 (87.0–157.0)	111.0 (69.0–155)	0.142	0.039
Fasting glucose, mg/dL	97.0 ± 13.5	98.4 ± 11.2	97.6 ± 13.2	0.650	0.698
Statin use	3480 (43.7)	107 (59.8)	25 (45.5)	0.0610	< 0.001
Aspirin use	4086 (51.1)	106 (61.2)	30 (54.6)	0.377	0.025
No. antihypertensive agents	2.0 ± 1.0	2.0 ± 1.0	2.2 ± 1.0	0.134	0.003
Randomization to intervention group	4034 (50.4)	75 (41.9)	18 (32.7)	0.224	0.003

Abbreviations: BMI, body mass index; BP, systolic blood pressure; CVD, cardiovascular disease; eGFR, estimated glomerular filtration rate; HDL, high density lipoprotein; MI, myocardial infarction; RMI, recognized MI; SD, standard deviation; SIQR, interquartile range; SMI, silent myocardial infarction; UACR, urinary albumin to creatinine ratio.

^a^ *p* value for comparison between SMI and RMI with the unpaired Student *t* test and *χ*2 for continuous and categorical variables, respectively.

^b^ *p* value for comparison among the three groups using ANOVA and *χ*2 for continuous and categorical variables, respectively.

There was a total of 37 SMI events among 4115 (0.89%) participants in the standard arm and 18 SMI events among 4127 (0.43%) intensive arm participants (1.1 vs. 2.3 cases per 1000 person‐years, hazards ratio [HR], 0.48; [95% confidence interval (CI), 0.27–0.84]; *p* value = 0.01) for a risk reduction of 52%. There were no differences between the strength of the association of intensive BP control on lowering the risk of SMI and RMI (Table 2). There were total of 104 RMI events among 4115 (2.5%) participants in the standard arm and 75 RMI events among 4127 (1.8%) intensive arm participants (4.6 vs. 6.5 cases per 1000 person‐years, hazards ratio [HR], 0.71; [95% confidence interval (CI), 0.52–0.95]; *p* value = 0.02) (Table 2).

**TABLE 2:** Effect of intensive versus standard treatment on incident SMI and RMI in SPRINT.

Treatment arm	Number of participants	Number of events	Events per 1000 person‐years	HR (95% CI)	*p*	*χ* ^2^ *p* for MI subtype difference^a^
Effect of intensive versus standard treatment on incident SMI						
Intensive	4127	18	1.1	0.48 (0.27–0.84)	0.01	0.23
Standard	4115	37	2.3			
Effect of intensive versus standard treatment on incident RMI						
Intensive	4127	75	4.6	0.71 (0.52–0.95)	0.02	
Standard	4115	104	6.5			

*Note:* Clinical site at randomization was used as a stratification factor.

Abbreviations: RMI, recognized myocardial infarction; SMI, silent myocardial infarction; SPRINT, systolic blood pressure intervention trial.

^a^ Lunn‐McNeil method was used to test whether intensive blood pressure control was associated with a differential lower risk for silent versus recognized MI.

**FIGURE 1:**
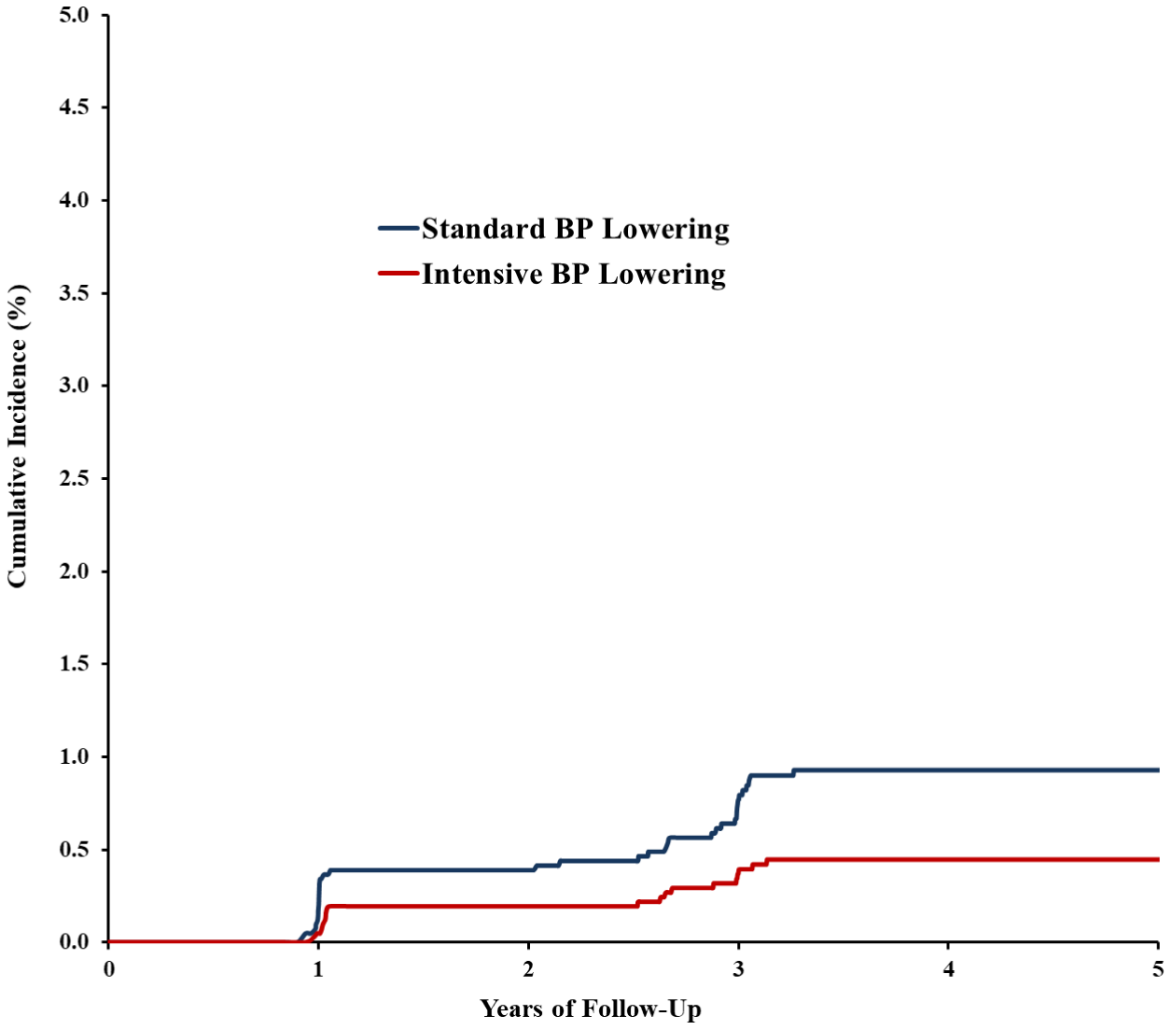
Cumulative incidence rate of silent myocardial infarction by treatment group.

The separation between treatment arms in the incidence of SMI events was apparent at 1 year (Figure 1). The rates of RMI incidence, categorized by SBP treatment arms, are depicted in Figure S1.

Due to the violation of the proportionality assumption for RMI as an outcome, the overall maximum follow‐up period of 5 years was subdivided into two categories: a short‐term follow‐up period of < 2.5 years and a long‐term follow‐up period ≥ 2.5 years. During the short‐term follow‐up, 58 RMI events occurred among 125 (46.4%) participants in the standard arm, and 52 RMI events occurred among 96 (54.1%) intensive, HR (95% CI): 1.19 (0.81–1.75), *p* = 0.35. During the long‐term follow‐up, there were 46 RMI events among 3990 (1.1%) participants in the standard arm and 23 RMI events among 4031 (0.57%) intensive, HR (95% CI): 0.58 (0.29–0.80), *p* = 0.004.

**FIGURE 2:**
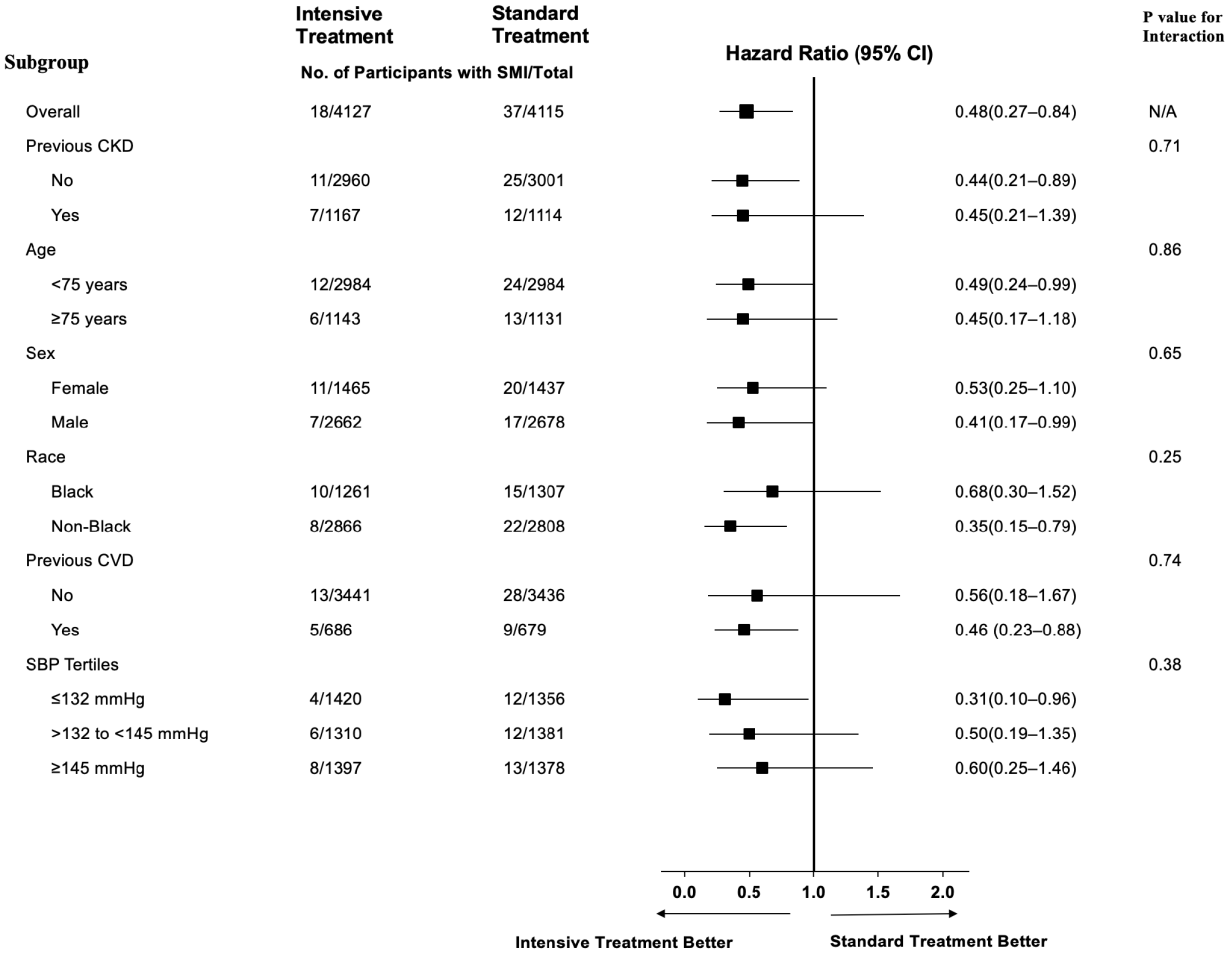
Effect of intensive blood pressure (BP) lowering on the risk of incident silent myocardial infarction (SMI) in prespecified SPRINT subgroups. CKD, chronic kidney disease; CVD, cardiovascular disease; SBP, systolic blood pressure.

While the effect of intensive SBP treatment on the risk of SMI events remained consistent across predefined subgroups, a more significant reduction in the risk of SMI was observed among individuals without a history of CKD, without prior CVD, non‐Black participants, and those with baseline SBP in the lowest tertile (Figure 2).

## 4 Discussion

In this post hoc analysis of the SPRINT trial, intensive BP control with the goal of SBP of less than 120 mmHg compared to the standard goal of SBP of less than 140 mmHg resulted in a significantly lower rate of SMI. While the benefits of intensive BP control in lowering the risk of SMI were consistent among prespecified SPRINT subgroups, the impact was more pronounced among non‐Black individuals, those with no prior CVD or CKD, and those with lower SBP. As expected, intensive BP control also resulted in a lower relative risk of RMI. There was no difference between the strength of the association of intensive BP control on lowering the risk of SMI and RMI. Hence, the benefit of intensive treatment of hypertension extend beyond preventing clinically manifested myocardial infarction (MI) to also encompass the prevention of silent or “subclinical” MI. To the best of our knowledge, this is the first large trial in which the benefits of intensive BP control to reduce the risk of SMI are demonstrated.

Hypertension is one of the major modifiable risk factors for RMI and SMI. In a 30‐year follow‐study of Framingham, the proportion of SMI detected by ECG in participants without preexisting heart disease increased from 18.5% among normotensive men to 35.4% among men with hypertension. For women, this proportion increased from 27.8% to 44.6% (Kannel, Dannenberg, and Abbott 1985). Similarly, in Iceland's MI study, midlife CVD risk factors in midlife were associated with both SMI detected by CMR, and SBP was the most significant risk factor associated with SMI (McAreavey et al. 2016). Further, an analysis of the REasons for Geographic and Racial Differences in Stroke (REGARDS) study found that participants with SMI were less likely than those with RMI to use aspirin, beta‐blockers, statins, and angiotensin‐converting enzyme inhibitors (Levitan et al. 2013). These findings highlight the missed opportunities that likely contribute to the elevated risk associated with UMI. Furthermore, consistent with a prior analysis among participants in the Atherosclerosis Risk in Communities (ARIC) study (Zhang et al. 2016), in the current study we observed some differences in the prevalence of SMI based on sex and race/ethnicity. The underlying reasons behind these differences remain unclear and require further investigation for elucidation.

SMI is a well‐established predictor of coronary heart disease (CHD), heart failure (HF), sudden cardiac death (SCD), ischemic stroke, and mortality (Brunetti et al. 2019; Merkler et al. 2021; Vähätalo et al. 2019). A recent comprehensive meta‐analysis found that SMI detected by ECG or cardiac magnetic resonance imaging (CMR) was associated with all‐cause mortality and multiple CVD outcomes with risks comparable to those with RMI (Yang et al. 2020). CMR is a highly sensitive and specific modality to detect SMI and has consistently improved the prediction of CVD and mortality, while utilizing ECG to screen SMI has low sensitivity but high specificity (Yang et al. 2020; Schelbert, Iyer, and Miller 2020). Further, ECG may add additional predictive value for mortality and CVD events, but results have been inconsistent (Soliman 2019; Yang et al. 2020). Therefore, the United States Preventive Services Task Force (USPTS) concluded that there is insufficient evidence to assess the balance of benefits and harms of ECG screening to prevent CVD events in asymptomatic individuals at intermediate or higher risk of CVD (Curry et al. 2018). However, the American Heart Association and American College of Cardiology consider ECG screening “reasonable” in asymptomatic individuals with hypertension or diabetes and stated that ECG “may be considered” in asymptomatic individuals without hypertension or diabetes (Greenland et al. 2010). The 2021 European Society of Cardiology guidelines on cardiovascular prevention recommends a 12‐lead ECG for all hypertensive individuals to detect hypertension‐mediated organ damage (Class 1, Level B) (Visseren et al. 2021).

While routine CMR may be cost‐prohibitive, ECG surveillance of elderly individuals with hypertension and those at higher cardiovascular risk may be warranted as preventive strategies may reduce the risk of future SMI. The increasing prevalence of SMI with age, with some reports suggesting the prevalence of SMI exceeds the prevalence of RMI with approximately 1–2 additional SMI for every RMI in the elderly population, further supports the consideration of screening for SMI in carefully selected populations (Schelbert et al. 2012). Further studies are needed to explore how to integrate ECG or imaging modalities to detect myocardial ischemia in such high‐risk populations.

Certain limitations of the study are the following: First, overall, fewer SMI events due to strict criteria of only the presence of a new major Q wave likely limited the statistical power of the analysis to detect any subgroup differences. Second, fewer SMI events also resulted from the inherent limitation of ECG as a low‐sensitive method compared to highly sensitive modalities such as CMR. Therefore, we may have missed many cases of SMI in both treatment arms without any risk of misclassification. Third, our results may not be generalizable to other populations who did not meet eligibility criteria for SPRINT, such as individuals with diabetes mellitus, individuals with low CVD risk, prior stroke, < 50 years old, and individuals residing in nursing homes or assisted living facilities. The strengths of our study include a large sample size from the well‐designed clinical trial of a diverse population at high risk of CVD. The data variables were collected using standardized procedures in the context of a clinical trial with the central reading of ECGs blinded to the treatment assignment.

## 5 Conclusions

In participants at high risk for cardiovascular events, targeting an SBP of < 120 mm Hg, as compared with < 140 mm Hg, significantly reduced the risk of developing SMI by 52%. This benefit was similar across all predetermined subgroups. There were no differences between the strength of the association of intensive BP control on lowering the risk of SMI and RMI. Therefore, the benefit of intensive treatment of hypertension extend beyond preventing RMI to also encompass the prevention of SMI.

## Author Contributions

Conceptualization, methodology, reviewing, and editing: Muhammad Imtiaz Ahmad, Richard Kazibwe, and Elsayed Z. Soliman; original draft preparation and statistical analysis: Muhammad Imtiaz Ahmad; administrative, technical, or material support: Elsayed Z. Soliman; supervision: Elsayed Z. Soliman; reviewing and editing: Elsayed Z. Soliman, Sanjay Singh, and Lin Y. Chen, Muhammad Imtiaz Ahmad takes full responsibility for the work and the conduct of the study.

## Ethics Statement

A de‐identified dataset was obtained from the National Heart, Lung, and Blood Institute's Biologic Specimen and Data Repository Information Coordinating Center after the institutional review board at the Medical College of Wisconsin approved the study.

## Consent

All participants provided written informed consent for participation in the SPRINT trial. The trial was approved by the institutional review board at each site and was registered with ClinicalTrials.gov.

## Conflicts of Interest

Elsayed Z. Soliman is an Editorial Board member of Annals of Noninvasive Electrocardiology and a co‐author of this article. To minimize bias, they were excluded from all editorial decision‐making related to the acceptance of this article for publication. The other authors declare no conflicts of interest.

## Supporting information


Figures S1–S2.

## Data Availability

All the data used in this analysis are available in the National Heart, Lung, and Blood Institute BioLINCC repository, https://biolincc.nhlbi.nih.gov/studies/sprint/.
