# Supplementary material for: Effect of Intensive Blood Pressure Lowering on the Risk of Incident Silent Myocardial Infarction: A Post Hoc Analysis of a Randomized Controlled Trial
Source: Ann Noninvasive Electrocardiol. 2024 Oct 3;29(6):e70018. doi: 10.1111/anec.70018 (PMC11447273; doi:10.1111/anec.70018)

**Supplementary Material**

**Effect of Intensive Blood Pressure Lowering on the Risk of Silent Myocardial Infarction: A Post hoc Analysis of a Randomized Controlled Trial**

**Supplemental Figure 1.** Cumulative Incidence Rate of Recognized Myocardial Infarction by Treatment Group

**Supplemental Figure 2**. CONSORT Diagram for the Systolic Blood Pressure Intervention Trial (SPRINT)

**Supplemental Figure 1.** Cumulative Incidence Rate of Recognized Myocardial Infarction by Treatment Group


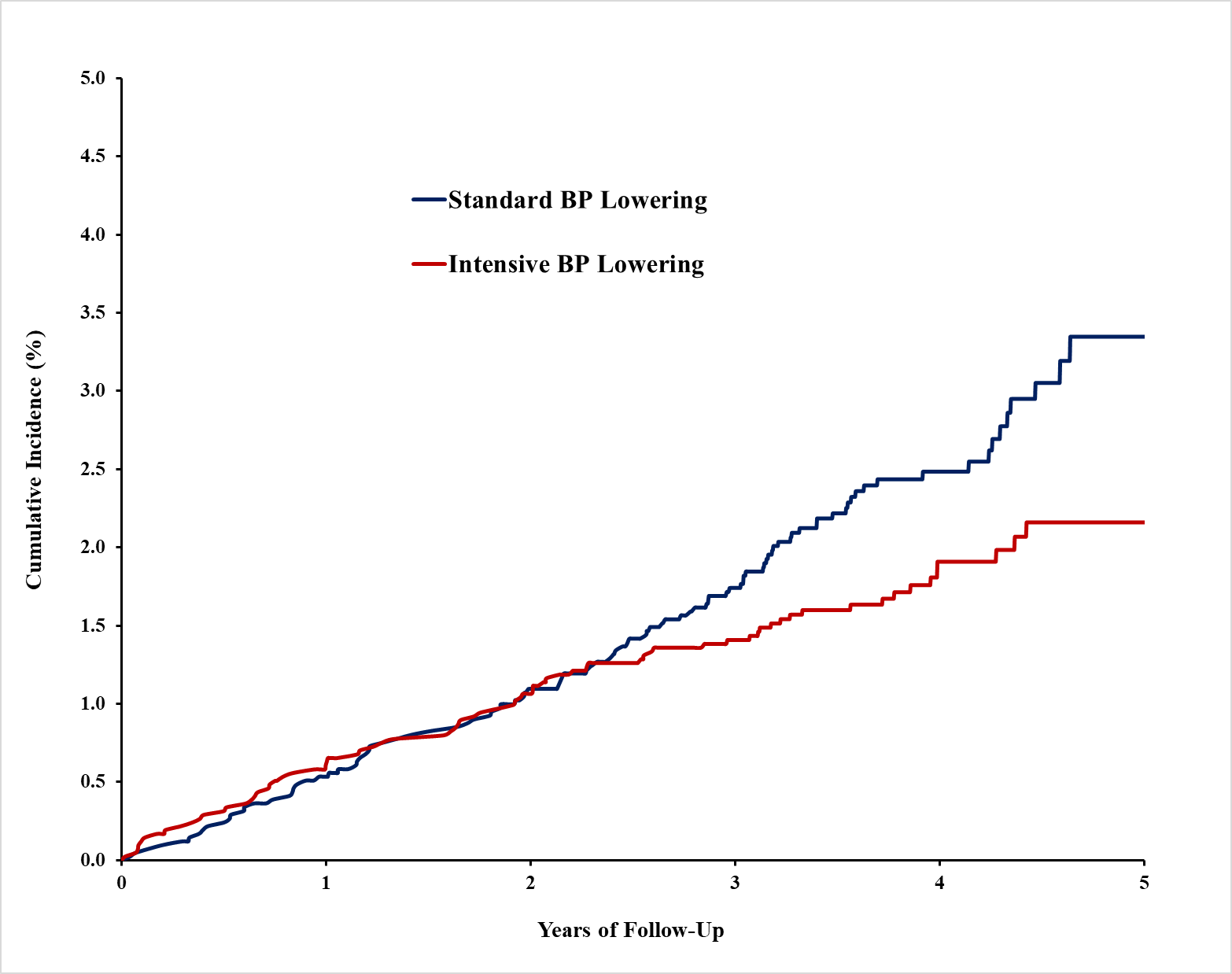


**Supplemental Figure 2**. CONSORT Diagram for the Systolic Blood Pressure Intervention Trial (SPRINT)


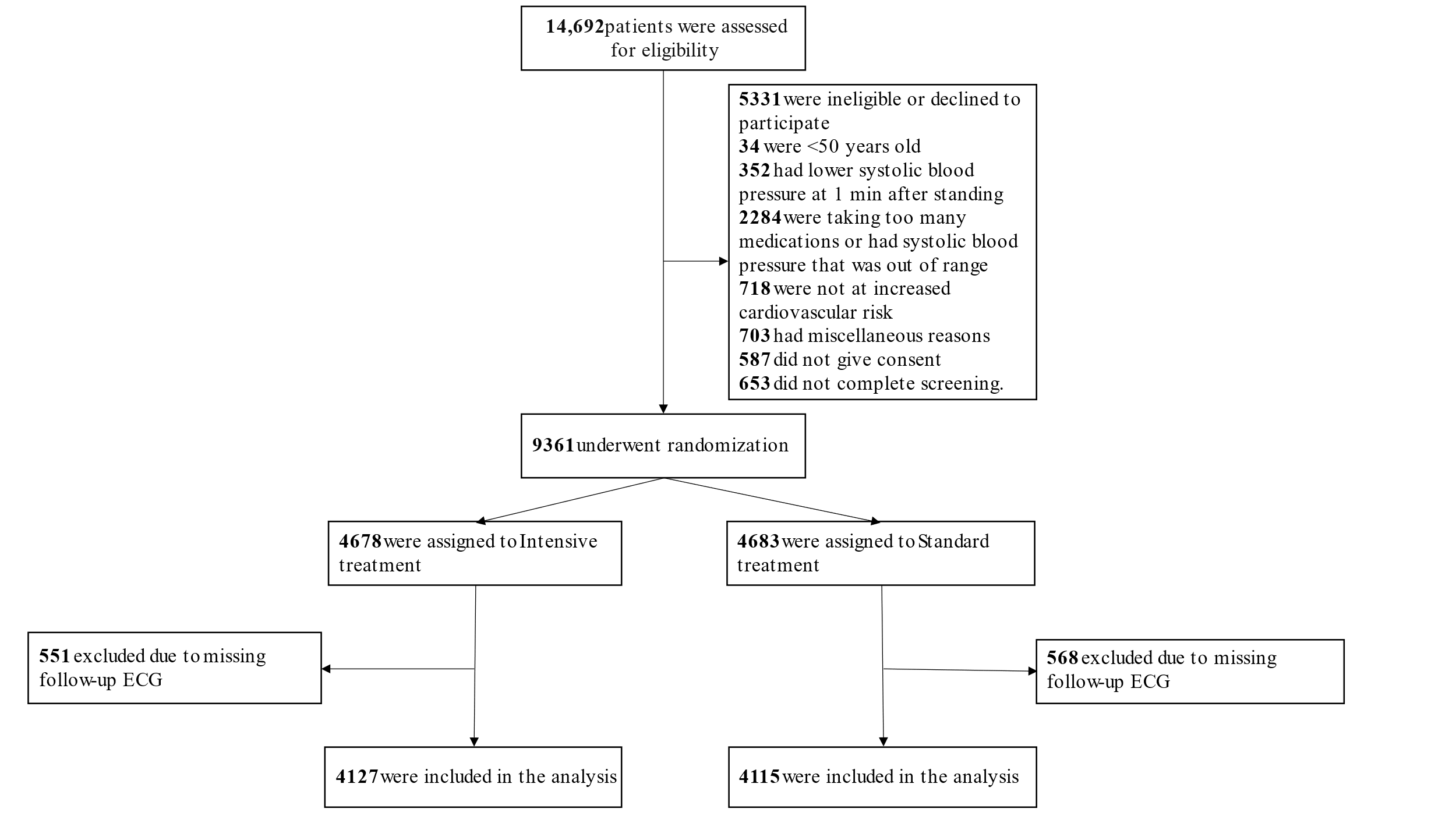

Supplement: Supplementary file 1 — Figures S1–S2. [file ANEC-29-e70018-s001.docx]
